# Promising Radiative Cooling Materials and Their Application in Construction and Building

**DOI:** 10.3390/polym18050596

**Published:** 2026-02-28

**Authors:** Chaoqun Ji, Biyu Li, Kaisheng Zeng, Yonghao Ni, Jianguo Li, Ruiying Zhang, Bin Chen

**Affiliations:** 1 School of Materials Engineering, Fujian Agriculture and Forestry University, Fuzhou 350108, China; jichaoqun999@163.com (C.J.); 19126548602@163.com (B.L.); z2069720683@163.com (K.Z.); jianguolicn@fafu.edu.cn (J.L.); 2 Department of Chemical Engineering, University of New Brunswick, Fredericton, NB E3B 5A3, Canada; yonghao@unb.ca

**Keywords:** thermal radiation, solar reflectivity, radiative cooling, building and construction

## Abstract

Radiative cooling technology, which leverages the emission of long-wave infrared radiation to deep space, offers a promising passive cooling solution that can reduce the energy consumption associated with conventional air conditioning systems. This technology is particularly relevant in tropical and subtropical regions, where buildings are exposed to high levels of solar radiation and excessive heat. Passive radiative cooling materials, such as petroleum-, inorganic- and cellulose-based materials, have shown significant potential in reducing building temperatures (more than 8 °C at daytime and 10 °C at nighttime) and enhancing energy efficiency by weakening the utilization of air conditioning. This review explores the development of promising radiative cooling materials, focusing on their raw materials, manufacturing, and key distinction (such as high solar reflectivity of >90% and middle-infrared band light emissivity of >0.9) for radiative cooling. Further, the progressive application of radiative cooling material in building and construction is significantly discussed, focusing on the cooling performance, mechanical properties, hydrophobicity and long-term stability. Lastly, future directions for advancing radiative cooling materials for building applications are presented, emphasizing the importance of integrating sustainability, up-scale manufacturing, and low cost with high thermal management performance.

## 1. Introduction

Solar radiation, as the main energy source for life on Earth, not only provides a basic guarantee for human survival and development, but also provides indispensable energy for plants and animals. Although solar radiation offers abundant energy to Earth’s ecosystem, excessive radiation can trigger negative effects, especially in tropical and subtropical regions. In these areas, buildings are often subjected to continuous heating from strong solar radiation, resulting in a significant increase in indoor temperature, which in turn causes discomfort and seriously affects the quality of life [1,2,3]. To cope with the pain caused by high temperatures, air conditioning systems have been widely adopted and have rapidly evolved into a technology used worldwide. However, as energy-intensive technologies, air conditioning systems cannot be ignored for their environmental impact. According to recent projections by the International Energy Agency (IEA), global peak electricity demand is expected to rise by approximately 40% by 2035, a trend largely driven by surging demand for space cooling. This rapid growth not only exacerbates the global energy crisis but also puts greater pressure on the environment [4]. Therefore, it is urgent to take effective measures to mitigate the energy crisis and the aggravation of environmental pollution.

**Figure 1:**
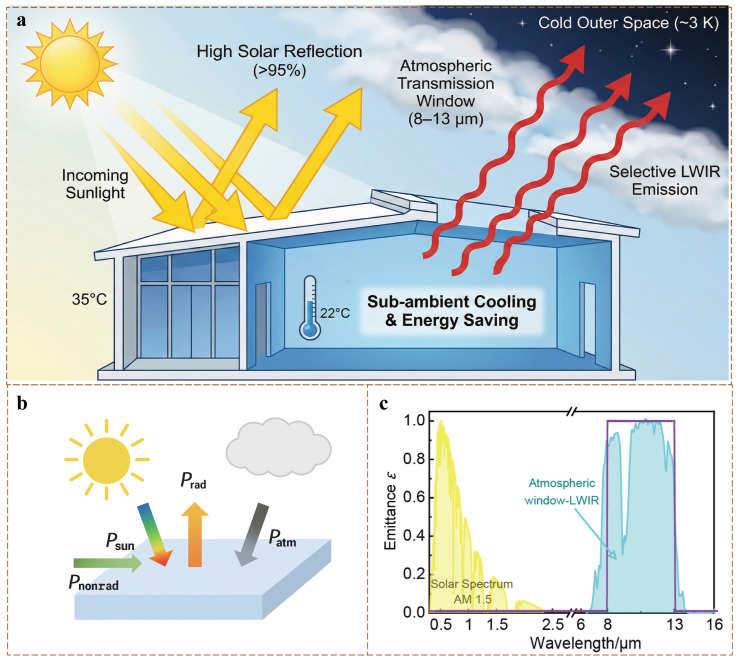
(**a**) Schematic illustration of the working mechanism for passive daytime radiative cooling in building applications. The cooling surface achieves sub-ambient temperatures and building energy savings by operating on a dual mechanism: simultaneously maximizing solar reflection to minimize external heat gain, and selectively emitting long-wave infrared radiation (LWIR) through the atmospheric transmission window (8–13 μm) into the cold deep space (~3 K). (**b**) Simplified diagram of the energy flow on the Earth’s surface. (**c**) Schematic diagram of the solar spectrum and the terrestrial radiation spectrum [14].

Meanwhile, as the concept of sustainable development gains popularity, more research is focusing on the development of low- or zero-energy cooling technologies. Against this backdrop, passive radiative cooling technology, as a promising cooling strategy, has gradually become a hot topic in this research field. Passive radiative cooling technology achieves self-cooling below ambient temperature without requiring any external energy input. Fundamentally, as schematically illustrated in Figure 1a, it operates on a dual mechanism: simultaneously reflecting incident solar radiation to minimize heat gain and emitting internal heat into the cold deep space (approximately 3 K) through the atmospheric transmission window (8–13 μm) via long-wave infrared radiation [5,6]. Therefore, it offers significant advantages in energy conservation and emissions reduction, effectively alleviating the energy consumption problem caused by air conditioning systems.

Based on this principle, researchers have proposed and designed a variety of organic and inorganic materials to improve radiative cooling efficiency and expand their application range [7,8,9,10]. These new materials not only have high thermal radiation capacity but also can reflect solar radiation to the maximum extent, thereby improving overall thermal management efficiency. By optimizing the optical properties and thermal conductivity of the materials, high-performance thermal management materials have been successfully developed. These materials can play a stable role in radiative cooling under variable environmental conditions, greatly reducing the energy demand of traditional cooling methods such as air conditioning [11,12,13].

To establish a clear theoretical foundation, Figure 1a illustrates the fundamental working mechanisms and optical requirements of passive daytime radiative cooling (PDRC). As shown in Figure 1a, PDRC relies on a dual mechanism to achieve sub-ambient cooling in buildings. First, the surface must highly reflect incoming sunlight (0.3–2.5 μm) to minimize heat gain. Second, it must strongly emit internal heat into cold outer space (~3 K) through the atmospheric window (8–13 μm, Figure 1c). Discussing these mechanisms prior to introducing specific materials is essential, as it establishes the core optical criteria for PDRC. This perfectly aligns with the aim of this review: evaluating how various materials (inorganic, polymer, and cellulose) fulfill these physical requirements for building thermal management. To further quantify this cooling capability, the specific energy balance of the cooling surface is detailed in Figure 1b.

Figure 1b presents a simplified description of the energy flow at the Earth’s surface. For a terrestrial surface exposed to the atmosphere, the net radiative effect is a combination of the following components:

$$P_{cooling}=P_{rad}\left(T\right)-P_{sun}-P_{atm}(T_{amb})-P_{non-rad}$$

Here, $P_{rad}(T)$ represents the thermal radiation power emitted by the material surface at temperature T; $P_{sun}$ denotes the solar radiation power absorbed by the surface; $P_{atm}(T_{amb})$ refers to the atmospheric radiation power absorbed by the surface at an ambient temperature of $T_{amb}$; and $P_{non-rad}$ indicates the non-radiative heat transfer power, which includes contributions from thermal convection and thermal conduction.

Research indicates that the heat transfer coefficient significantly influences both the net cooling power and the maximum cooling performance. Therefore, effectively minimizing thermal conduction and thermal convection is crucial in radiative cooling (RC) systems. Under clear atmospheric conditions, the atmosphere exhibits high transmittance in the 8–13 μm range, which overlaps with the peak radiation spectrum of a blackbody at 300 K [14]. This atmospheric window coincides with the wavelength range of thermal radiation emitted by surface objects. Within this window, radiative cooling materials radiate heat from the Earth’s surface into space, thereby facilitating energy dissipation. Consequently, significant atmospheric absorption primarily occurs outside the bandwidth of this window.

This paper reviews the main radiative cooling materials, including inorganic nanoparticles, organic polymers, and natural cellulose. It focuses on the preparation, performance, advantages, and disadvantages of these materials. Furthermore, building applications represent a significant window for the application of radiative cooling materials, as building air conditioning consumes 16% of the world’s energy. The paper analyzes the radiative cooling performance, mechanical strength, hydrophobicity, and thermal insulation properties of radiative cooling materials used in buildings. Finally, this paper explores the development directions of inorganic nanoparticles, organic polymers, and natural cellulose materials in the field of radiative cooling and provides guidance on green radiative cooling materials for buildings.

## 2. Radiative Cooling Materials

### 2.1. Inorganic Radiative Cooling Materials

Many common phenomena in nature are closely related to radiative cooling: for example, on clear nights, even if the temperature is above zero, plant leaves may still have dew or frost; and a major reason for the large diurnal temperature range in desert regions is the radiative cooling effect of the sky, which causes the ground temperature to drop sharply at night. Research on radiative cooling primarily focuses on the thermal performance of coolers and systems, with coolers as the primary research object. Radiative cooling is mainly divided into two modes: nighttime cooling [15,16,17] and daytime cooling [18,19,20]. In the earliest studies, inorganic radiative cooling materials [21,22,23,24] were mainly used for nighttime radiative cooling, primarily because nighttime radiative cooling does not require high spectral selectivity of the cooler in the solar radiation band, making it relatively easy to implement. For example, Ma et al. [25] and Chae et al. [26] developed a series of silicon nitride (Si_3_N_4_)-based inorganic coatings for efficient radiative cooling. Meanwhile, early pioneering work by Granqvist and colleagues focused on silicon monoxide (SiO) coatings [17,21] and silicon oxynitride (SiO_x_N_y_) coatings [23] to achieve high emission characteristics of the cooler in the ‘atmospheric window’ band. Berdahl and Fromberg [27] used a magnesium oxide (MgO) ceramic coating as an infrared spectroscopy-selective cooler to achieve passive thermal management at 20 °C. They analyzed the potential of lithium fluoride (LiF) as an infrared spectroscopy-selective radiation-cooling material. Gentle and Smith [24] at the University of Technology Sydney doped silicon carbide (SiC) and silicon dioxide (SiO_2_) nanoparticles into a polyethylene film, resulting in a composite material with high emissivity in the atmospheric transparency window. Catalanotti et al. [28] prepared a spectrally selective radiative cooling material by adding a polyvinyl fluoride (PVF) film to an evaporated aluminum plate, which has good sky radiative thermal management performance. Although inorganic radiative cooling materials can achieve good radiative thermal management at night, they still have limitations in providing a sufficient cold source during the day.

**Figure 2:**
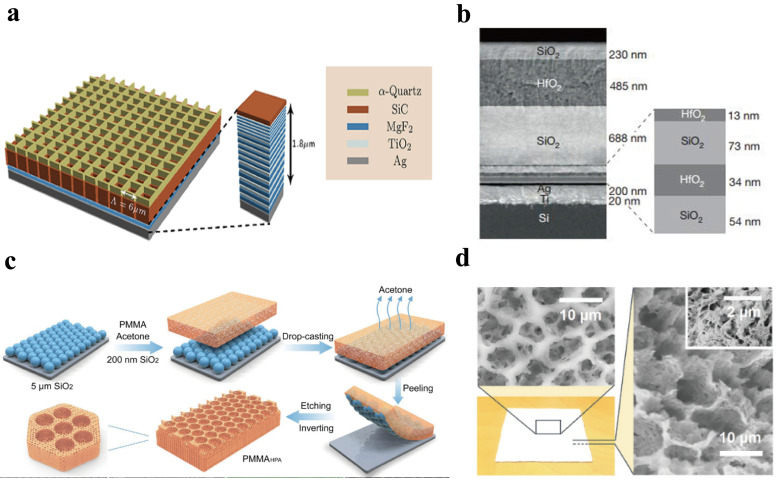
(**a**) Optimized daytime radiative cooler design [29]. (**b**) SEM image of a seven-layer alternating structure [33]. (**c**) Schematic illustration of the synthesis of porous poly (methyl methacrylate) (PMMA) thin film [18]. (**d**) Micrographs showing top and cross-section views of P(VdF-HFP) HP. The inset shows the nanoporous features [34].

Compared with nighttime radiative cooling, it is more difficult to achieve radiative cooling below ambient temperature during the day because solar radiation power is much greater than radiative cooling power, and even absorbing a small amount of solar radiation will significantly affect the cooling effect. However, with the development of micro and nanomaterials, daytime radiative cooling technology has made breakthroughs. The new generation of coolers not only has high emissivity in the “atmospheric window” band but also has extremely strong solar reflectivity, enabling them to achieve passive cooling below ambient temperature under daytime solar radiation conditions. The new generation of inorganic radiative cooling materials mainly includes integrated photonic crystals [29,30], metamaterials [31], thin films and coatings [32], etc. Rephaeli et al. [29] proposed an ultra-wideband photonic structure that can achieve daytime radiative cooling calculation results. As shown in Figure 2a, the cooler consists of two square pores made of SiC and quartz with periodically etched two-dimensional photonic crystal layers, providing surface phonon-polariton resonances in the 8–13 μm range as a selective emitter in the atmospheric window. At the bottom is a solar reflector made of a one-dimensional photonic crystal, whose reflection spectrum covers the entire solar spectrum. Under optimized conditions, the emitter can achieve a net cooling power exceeding 100 W/m^2^ under direct sunlight.

To fully optimize the spectral characteristics of the radiative cooler across the entire wavelength range, the team led by Fan Shanhui at Stanford University [33] designed a metal-dielectric photonic crystal radiative cooler (as shown in Figure 2b). This cooler consists of an emitting layer composed of hafnium oxide (HfO_2_) and silicon dioxide (SiO_2_) of different thicknesses, and a reflective layer comprising silver (Ag). In the solar spectral band, its reflectivity can reach 0.97, while in the atmospheric transparency window, the average emissivity is about 0.65. The photonic crystal cooler was tested on the roof of Stanford University in California. The results showed that under solar irradiance of 800–870 W/m^2^ during the day, the cooler surface can still achieve a low temperature of 4.9 °C below the surrounding environment. According to theoretical speculation, the cooler can withstand solar radiation during the day.

Significant progress has been made in nanophononics research, resulting in a large number of daytime radiation-radiative cooling materials with promising applications. While these designs demonstrate high efficiency in radiation cooling, they are typically expensive and complex to fabricate. They are primarily manufactured on a small scale using photolithography or electron-beam evaporation, which requires bulky casings and shielding. Furthermore, because most photonic materials are semi-transparent, they must be deposited on metallic mirrors to achieve high solar reflectivity. Therefore, the search for more direct methods and more scalable radiation-radiative cooling materials is essential.

### 2.2. Petroleum-Based Radiative Cooling Materials

Petroleum-based polymers [35,36,37,38,39] have seen rapid development in radiation cooling technology due to their good optical properties and economic practicality. Petroleum-based radiative cooling materials are diverse, including porous polymer films, selective thermal radiation composite films, and super fabric materials. Wang et al. [18] reported a layered polymethyl methacrylate (PMMA) film with a combination of micropore arrays and random nanopores for efficient passive radiation cooling during the day and night. This layered porous array PMMA film exhibits sufficiently high solar reflectivity (95%) and excellent long-wave infrared thermal emissivity (0.98), achieving a cooling temperature reduction of 8.2 °C at night and a cooling temperature reduction of 6.0~8.9 °C during the day, with an average cooling capacity of 85 W/m^2^ at noon. Even in hot, humid climates with solar intensity of 930 W/m^2^ and relative humidity of 64%, the cooler can achieve a cooling of 5.5 °C. Mandal et al. [34] replaced dielectric materials, such as zinc oxide in cold roof coatings, with pores that scatter air. They developed an inexpensive, layered, porous polymer poly(vinylidene fluoride-co-hexafluoropropylene) (P(VdF-HFP) HP) coating for daytime radiative cooling. P(VdF-HFP) HP inherently absorbs negligible amounts of sunlight, producing multiple emission peaks due to the different vibrational modes of its molecular structure. As a result, the coating exhibits excellent selectivity (solar reflectance 0.96, infrared emissivity 0.97) and wide-angle uniformity, eliminating the need for metal reflectors.

Furthermore, P(VdF-HFP) HP also exhibits good weather resistance, anti-fouling properties, and UV radiation resistance. When placed outdoors, the coating retains its optical properties almost unchanged for extended periods. To enable the radiative cooling materials to have good hydrophobic properties, Huang et al. [40] developed a layered polyvinylidene fluoride/polydimethylsiloxane (PVDF/PDMS) porous film, which has high solar reflectivity (97%), high thermal infrared emissivity (96%) and strong superhydrophobic properties, with a contact angle of 160.2°. The synergistic effect of effective solar reflection and thermal infrared emission enables the film to generate a sub-ambient temperature drop of 12.3 °C under strong sunlight, and in practical applications, it provides good cooling efficiency for automobiles. More importantly, the superhydrophobicity keeps the film free of contamination through self-cleaning, maintains good radiative thermal management performance, and allows outdoor use for a long time.

**Figure 3:**
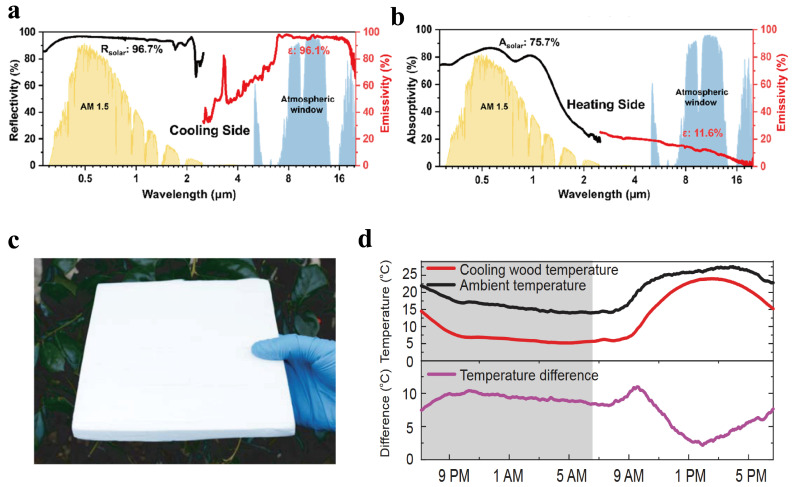
(**a**) Measured UV−vis−NIR reflectivity (black line) and infrared emissivity (red line) of the cooling side of the dual-mode film [42]. (**b**) Measured UV−vis−NIR absorptivity (black line) and infrared emissivity (red line) of the heating side of the dual-mode film [42]. (**c**) Cooling wood [44]. (**d**) Performance of Cooling wood [44].

Li et al. [41] developed a layered polymer nanofiber film by electrospinning, which can selectively emit mid-infrared light and effectively reflect sunlight, thus exhibiting excellent all-day radiative thermal management performance. Among them, the chemical functional groups C-O-C (1260~1110 cm^−1^) and C-OH (1239~1030 cm^−1^) of the nanofiber film have a selective emissivity of 78% in the atmospheric window wavelength range of 8~13 μm, and the nanofibers with controllable diameter have a high reflectivity of 96.3% in the wavelength range of 0.3~2.5 μm. Therefore, compared with the non-selective emitter, the cooling efficiency of this selective thermal emitter is improved by ~3 °C at night and by 5 °C under daytime illumination. Shi et al. [42] demonstrated an all-day dual-mode film by decorating porous polyvinylidene fluoride with nitride nanosheets and then using phase inversion to obtain a rich coral-like layered structure. The cooling side of the bimodal film exhibits high solar reflectivity (96.7%) and infrared emissivity (96.1%). Therefore, the daytime radiative cooling temperature is 9.8 °C, with a theoretical cooling power of 107.5 W/m^2^, while the nighttime temperature drop is 11.7 °C, with a theoretical cooling power of 140.7 W/m^2^. Meanwhile, as shown in Figure 3a,b, the heating side of the dual-mode film has lower infrared emissivity (11.6%) and higher solar absorptivity (75.7%), with a passive radiative heating capacity of 8.1 °C, and good active solar heating and Joule heating capabilities. The dual-mode film can be easily switched between cooling and heating modes by flipping to adapt to dynamic cooling and heating scenarios. While petroleum-based polymers exhibit exceptional radiative cooling capabilities, the conventional fabrication of their highly porous structures (such as energy-intensive freeze-drying) often entails a substantial carbon footprint. To mitigate this environmental impact, Peng et al. [43] recently developed a scalable, low-carbon foam-like aerogel incorporating synthetic melamine-formaldehyde resin. By employing a green ambient-drying process that completely bypasses complex freezing and solvent exchange, they significantly reduced the carbon emissions and energy consumption typically associated with manufacturing petroleum-derived aerogels. Despite this low-energy fabrication, the robust composite maintains optimized optical properties, with a high solar reflectance of 93% and an infrared emissivity of 94%, yielding a sub-ambient cooling drop of 4.8 °C. This strategy highlights a highly practical pathway to minimize the ecological footprint of traditional petroleum-based radiative coolers without sacrificing their thermal management performance.

### 2.3. Cellulose-Based Radiative Cooling Materials

As the most abundant natural polymer material in the world, lignocellulose has the advantages of being recyclable, biodegradable, and renewable. At the same time, due to the unique structure of lignocellulose and its high thermal radiation emissivity in the long-wave range of the “atmospheric window”, it has great application value for developing radiation-based cooling materials through the regulation of fiber structure (Table 1). In 2019, Li et al. [44] reported a cooling wood obtained through complete delignification and densification, whose mechanical strength can reach 8 times that of ordinary wood (as shown in Figure 3a,b). The nanofibers in the wood cause solar radiation to backscatter. At the same time, C-O in the cellulose structure (absorption concentrated at 1050 cm^−1^) makes the cooling wood have strong emission characteristics in the atmospheric transparency window, thus achieving a temperature lower than room temperature (>4 °C) and nighttime (>9 °C) during the midday period, with a maximum cooling power of up to 75 W/m^2^. Energy-saving estimates for radiative -cooled wood have found that up to 60% of energy can be saved. However, the thermal management performance of radiative -cooled wood is relatively low, and removing lignin from whole wood pieces is quite complex. The wide variety of wood types and structures also means the preparation process takes longer and requires more resources.

Despite the excellent optical properties and sustainability of natural cellulose, its inherent flammability poses a critical safety hazard for practical applications. To overcome this limitation without compromising cooling performance, researchers have developed flame-retardant cellulose composites through organic-inorganic hybridization. For instance, Chen et al. [45] successfully fabricated a robust cellulose fiber/SiO_2_ composite. The incorporation of inorganic SiO_2_ not only optimizes the hierarchical porous structure for enhanced solar scattering but also endows the composite with outstanding flame-retardant and antibacterial properties, making it highly reliable for scalable architectural deployment. Building on this concept of inorganic hybridization for fire safety, Zhao et al. [46] developed a highly compressible cellulose nanofibril (CNF) composite cryogel with exceptional flame-retardancy. By directionally assembling CNFs with hydroxylated boron nitride nanosheets (BNNS) and methyltrimethoxysilane via a freeze-casting method, they successfully created a stable interconnected network. The incorporation of inorganic BNNS and silane crosslinking not only preserves the anisotropic porous structure required for thermal insulation and radiative cooling, but also serves as a robust physical and thermal barrier, effectively suppressing flame propagation. This synergistic organic-inorganic structural design further validates that the severe fire hazards associated with natural cellulose can be successfully mitigated, paving the way for its safe deployment in practical building thermal management.

Cellulose acetate, a derivative of cellulose, is a thermoplastic resin obtained by esterification using acetic acid as a solvent and acetic anhydride as an acetylation agent under the action of a catalyst. Through processing and modification, it has seen rapid development in the field of radiative cooling materials. Chen et al. [47] introduced TiO_2_ nanoparticles, which possess advantages such as high refractive index, high infrared emissivity, and low solar absorption, into porous cellulose acetate films using an easy phase separation strategy. Due to solvent evaporation, the introduction of nano-TiO_2_ effectively improved the material’s solar reflectivity, reaching 0.97. Furthermore, the scattering model analysis showed that the high solar reflectivity was mainly due to the random aggregation of nanoparticles. These nanoclusters also contribute to the high infrared emissivity. The results showed that the film’s cooling capacity remained around 10 °C. The random nanocluster filling strategy avoids the complex processes associated with particles of specific sizes. However, TiO_2_ strong ultraviolet absorption will also affect the solar reflectivity of the radiative cooling materials. To overcome the poor recyclability and environmental concerns of traditional polymer coolers, Wei et al. [48] recently developed a fully recyclable ethyl cellulose (EC) coating via an eco-friendly non-solvent-induced phase separation (NIPS) method. The resulting hierarchical micro-nano porous structure, coupled with the strong intrinsic molecular vibrations of cellulose, enables a high solar reflectance of 0.96 and a mid-infrared emissivity of 0.96, achieving a sub-ambient cooling effect of 8.3 °C. Crucially, this EC coating demonstrates excellent compatibility with diverse building substrates (e.g., wood, glass, metals, and tiles) and can be repeatedly recycled using a simple ethanol/water system without degrading its cooling performance. This eco-friendly and highly compatible strategy provides a promising solution to the inherent limitations of conventional radiative cooling materials.

Similarly, to overcome the inherent hydrophilicity of natural cellulose and ensure its long-term environmental durability, Tian et al. [49] developed a superhydrophobic cellulose-fiber-based composite via an effective surface modification strategy. By air-spraying an ethanolic poly(tetrafluoroethylene) (PTFE) microparticle suspension onto the cellulose substrate, they constructed a dual-layered structure where PTFE particles are partially embedded within the microsized pores of the fibers. The introduction of low-surface-energy PTFE endows the composite with outstanding superhydrophobicity and self-cleaning capabilities. Consequently, this protective modification effectively prevents moisture penetration and surface contamination, ensuring stable and high-efficiency passive daytime radiative cooling performance under complex outdoor conditions while maintaining the material’s recyclability.

**Table 1:** Comparison of various radiative cooling materials.

Material Category	Representative System	Fabrication Technique	Solar Reflectivity (Rsolar)	IR Emissivity (εLWIR)	Cooling Performance (ΔT)
Inorganic	SiO on Aluminum [17]	Optical optimization/Deposition	High	High	~14 °C (Night)
	Photonic Crystals (SiC/Quartz) [29]	Photolithography/E-beam evaporation	High	High	
	Photonic Crystals (HfO_2_/SiO_2_/Ag) [33]	Alternating layer deposition	0.97	0.65	4.9 °C (Day)
Petroleum-based Polymer	Porous PMMA Film [18]	Phase inversion/Etching	0.95	0.98	6.0–8.9 °C (Day)
	Porous P(VDF-HFP) [39]	Layered porous coating	0.96	0.97	Sub-ambient (Day)
	Porous PVDF/MXene film [42]	phase inversion	0.96	0.97	9.8 °C (Day)
Cellulose-based Material	Cooling Wood [44]	Complete delignification & densification	≈0.96	>0.96	>4 °C (Day), >9 °C (Night)
	Cellulose Fibers/SiO_2_ [45]	Mechanical grinding & hot pressing	>0.96	High	6 °C (Day)
	Porous Cellulose Acetate/TiO_2_ [47]	Phase separation +1	0.97	High	~10 °C
	Ethyl Cellulose Coating [48]	Non-solvent-induced phase separation	0.96	0.96	8.3 °C (Day)

## 3. Building Applications of Radiative Cooling Materials

The radiative thermal management, waterproofing, thermal insulation, and mechanical properties of building materials are key factors in ensuring the comfort, energy efficiency, durability, and structural safety of buildings. Radiative thermal management performance helps buildings lower temperatures through high solar reflectivity and high infrared emissivity, reducing reliance on air conditioning systems, lowering energy consumption, mitigating the urban heat island effect, and improving building energy efficiency and living comfort. Waterproofing performance is crucial for preventing moisture penetration into the building structure, avoiding problems such as dampness, mold growth, and corrosion in walls, roofs, and basements, ensuring building durability and a healthy environment [50,51,52]. Thermal insulation helps maintain stable internal building temperatures, especially in hot climates, by blocking external heat from entering, reducing the burden on air conditioning systems. It also effectively insulates in cold environments, reducing heating energy consumption and improving building energy efficiency. Mechanical properties ensure that building materials can withstand various external loads, such as wind, ensuring structural stability and safety. Taking all these properties into account can optimize building energy efficiency, ensure long-term building stability, and promote the achievement of green building and sustainable development goals [53,54,55].

### 3.1. Radiative Cooling Performance

Radiative cooling materials reduce heat accumulation inside and outside buildings by effectively reflecting solar radiation and radiating heat into space, thereby significantly reducing air conditioning loads and energy consumption. During hot seasons, radiative cooling materials help buildings maintain a comfortable indoor temperature, enhancing comfort in living or working environments. Simultaneously, the application of radiative cooling materials helps alleviate the urban heat island effect and lower building temperatures [56,57,58,59].

**Figure 4:**
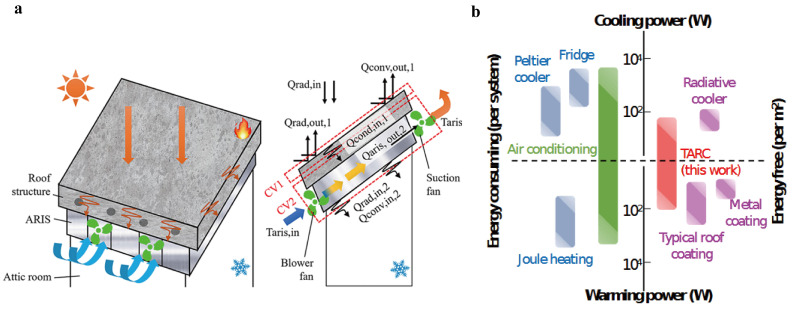
(**a**) Schematic diagram of the new cooling roof system [60]. (**b**) TARC in comparison to other thermal regulation systems, highlights the unique benefit of TARC of being simultaneously energy free and temperature adaptive [61].

Ho et al. [60] designed a novel cooling roof system. In an experiment in Kuala Lumpur, Malaysia, the application of white cooling paint reduced the roof’s exterior surface temperature by 22.8% compared to an exposed roof (Figure 4a). Lightweight foamed concrete (LFC) reduced the roof’s interior surface temperature from 50.50 °C to 45.36 °C, reducing solar energy absorption by approximately 10.18%. The introduction of hollow concrete slabs further reduced the roof’s interior surface temperature to 41.21 °C. The integrated cooling roof system, combining white cooling paint, LFC, hollow concrete slabs, and an active reflective insulation system (ARIS), reduced the roof’s interior surface temperature by 30.63%. After 5 h of intense sunlight, the average attic temperature was 33.86 °C, 8.03 °C lower than on an exposed roof.

Tang et al. [61] proposed a temperature-adaptive radiative coating (TARC) that adjusts its thermal emissivity based on ambient temperature, setting it to 0.20 below 15 °C and 0.90 above 30 °C. TARC provides efficient radiative cooling at high temperatures while reducing cooling at low temperatures, thereby avoiding overcooling (Figure 4b). Experiments show that TARC has a solar absorptivity of 0.25, making it suitable for a wide range of climates, especially in regions with large temperature differences. Simulation results show that TARC is more energy-efficient than existing roof coatings and can significantly reduce energy consumption and greenhouse gas emissions.

### 3.2. Waterproof Performance

Radiative-cooling building materials need to be hydrophobic to ensure long-term performance and durability. Moisture penetration can reduce the material’s heat reflection and infrared emissivity, thereby weakening its radiative cooling effect. Hydrophobic materials can prevent moisture adhesion, maintain their original high reflectivity and infrared emissivity, and ensure continuous, efficient radiative cooling. In addition, moisture intrusion can lead to corrosion, mold growth, or weathering of building materials, affecting the building’s appearance and function. Hydrophobicity can effectively prevent moisture erosion, extend the material’s service life, and reduce maintenance costs. At the same time, hydrophobic material surfaces are less prone to water accumulation or dirt adhesion, making them easier to clean, maintaining the material’s aesthetics, and avoiding the impact of stains on heat reflection performance. In conclusion, hydrophobicity is crucial for radiative-cooling building materials, improving durability, stability, and long-term performance, and ensuring excellent performance under various climatic conditions [62,63,64].

Xue et al. [65] reported a superhydrophobic P(VDF-HFP)/SiO_2_ composite film prepared by phase separation technology, designed to provide stable daytime radiative cooling for building exterior walls. In the study, the composite film was constructed with a micro-nano multilevel rough structure, resulting in a surface with excellent hydrophobic properties. The water contact angle reached 163 ± 2°, and the sliding angle was only 0.1 ± 0.1°, which means that pollutants such as rainwater and dust are difficult to adhere to the surface, thus ensuring that the film maintains high solar reflectivity and excellent infrared emission performance in outdoor environments for a long time. As a result, the film can effectively prevent the cooling effect from being reduced by environmental pollution in practical applications, playing an important role in energy conservation and ensuring an average temperature drop of about 10.3 °C under direct sunlight. Its superhydrophobic properties and radiative thermal management performance remain essentially unchanged after soaking in acid and alkali solutions and long-term UV irradiation, demonstrating its broad application prospects as a passive cooling material for building exterior walls.

Xu et al. [66] reported a superhydrophobic polytetrafluoroethylene/polyvinylidene fluoride (PTFE/PVDF) coating prepared by a one-click spraying method. This coating is designed for passive daytime radiative cooling and can be applied to building exterior walls to achieve energy-efficient cooling. Research data shows that the water contact angle of the coating is as high as 153.7° and the sliding angle is only 7.7°, which indicates that it has excellent hydrophobicity and self-cleaning ability, effectively preventing rainwater and dust pollution, maintaining a solar reflectivity of up to 93.7% and an infrared emissivity of 93.3%, thereby achieving a radiative cooling temperature of about −5.8 °C under direct sunlight (when shielding the convective heat effect). This excellent superhydrophobic performance not only ensures long-term stable operation of the coating but also provides a green, energy-free passive cooling solution for building exterior walls, helping reduce building cooling loads and energy consumption.

Similarly, to ensure long-term outdoor durability, Zhao et al. [67] developed a highly hydrophobic magnesium silicate hydroxide (MSH) coating using a PDMS binder. It achieves a water contact angle of 140.5°, providing excellent self-cleaning against rain and dust. Optically, the coating exhibits 94.4% solar reflectance and 97.6% mid-infrared emissivity, enabling an 8.2 °C sub-ambient temperature drop and 94.5 W/m^2^ cooling power under direct sunlight. Crucially, its robust hydrophobicity resists moisture penetration, maintaining superior cooling performance even under high humidity for building applications.

### 3.3. Thermal Insulation Performance

Building materials need to have good thermal insulation properties to ensure the comfort and durability of buildings under different climatic conditions. Thermal insulation can effectively prevent external heat from entering the room, especially in hot climates, helping avoid excessively high indoor temperatures caused by solar radiation and reducing the burden on air conditioning, while maintaining a comfortable indoor environment. In addition, thermal insulation materials can reduce heat loss in winter, help maintain a warm indoor temperature, and improve building energy efficiency [68,69,70].

Zhou et al. [71] reported a sustainable porous PDMS sponge prepared via a sugar-templating method. This material uses an environmentally friendly process (no toxic solvents, sugar is recyclable) to construct a structure with micron-sized pores at a cost of only about $17.1 per square meter. Optically, the material exhibits strong visible-light scattering (an absorption rate of 0.07 in the 350–750 nm band, a 42% reduction compared to the original PDMS) and efficient infrared radiation (an average emissivity of 0.96 in the 8–13 μm band), meeting the dual requirements for daytime radiative cooling. In terms of thermal performance, outdoor testing showed that it can achieve a temperature drop of 4.6 °C and a cooling power of 43.17 W/m^2^ under 980 W/m^2^ of solar irradiation, while also exhibiting excellent thermal insulation (thermal conductivity of 60) mW/(m·K). In practical applications, the interior temperature of a model house covered with this material is 3.7 °C lower than that of a traditional white painted roof.

Jiang et al. [72] developed a phase change radiation cooling material (PCRCM) based on PDMS foam and an octane phase change material, aiming to provide all-weather thermal management and energy-efficient solutions for building exterior walls. Research data shows that the average pore size of the original foam is approximately 224 μm. The micrometer-sized PCRCM is reduced to approximately 168 μm after phase change material (PCM) infusion, thereby maintaining the material’s porous structure and efficient scattering performance. In high-temperature cooling mode, the solar reflectivity of PCRCM reaches approximately 96%. In contrast, in low-temperature heating mode, it decreases to approximately 82.6%, effectively reducing indoor temperature and cooling load in summer, and preventing excessive cooling in winter. Outdoor tests show that the material can reduce summer temperatures by up to 17 °C and maintain a temperature 3–5 °C above ambient at night. Furthermore, Energy Plus model simulations show that, across different climatic conditions in China, using PCRCM for building exterior walls can significantly reduce summer cooling energy consumption and reduce additional winter heating needs, demonstrating its broad application prospects for building energy conservation.

Alongside polymer-based foams, advanced biomass-based foams also offer highly efficient thermal management solutions. For instance, Wang et al. [73] developed a thermal-insulating cellulose/wood foam (CWF) via a freeze-casting strategy. Its anisotropic porous structure acts as a robust thermal barrier to restrict downward heat conduction while maximizing photon scattering. Consequently, the CWF achieves an impressive 95.2% solar reflectance and 94.8% mid-infrared emissivity. Driven by these synergistic thermal insulation and passive radiative cooling capabilities, it delivers a theoretical cooling power of approximately 130 W/m^2^, providing a sustainable, eco-friendly alternative for passive-cooling building envelopes.

Particularly, to address the stringent structural morphology and thermal insulation requirements for architectural materials, silicate-based hollow structures—resembling the fundamental insulation mechanism of traditional foam glass—have emerged as exceptional thermal barriers. For instance, Sun et al. [74] developed a composite coating utilizing SiO_2_-coated hollow glass bubbles. The unique hollow morphology of these silicate micro-bubbles plays a dual role in thermal management. Optically, it alters the internal light path, facilitating intense backscattering of solar photons to block external heat gain at the source. Thermally, the enclosed hollow interior severely restricts downward thermal conduction pathways, acting as an intrinsic micro-scale thermal insulator. Furthermore, aided by its micro-/nano-hierarchical roughness, the coating achieves a superhydrophobic surface with a water contact angle of 157°. This exceptional moisture resistance prevents water from infiltrating and compromising the insulating air voids, maintaining its structural thermal resistance and achieving a remarkable ambient temperature drop of 11.1 °C under direct outdoor sunlight.

Building upon this concept of hierarchical silicate morphologies, Yang et al. [75] recently introduced a novel “pore-in-pore” structured composite foam. By constructing a three-dimensional self-supporting silicon dioxide (SiO_2_) network within the existing micropores of a polymer matrix, this advanced structural morphology serves as a highly effective thermal barrier. The encapsulated multi-scale pores not only maximize the scattering of solar radiation but also trap stagnant air to severely restrict downward heat transfer. This precise morphological engineering further exemplifies how integrating silicate-based porous networks can effectively replicate the insulation benefits of traditional foam glass, providing a robust and scalable solution for practical building thermal management.

### 3.4. Mechanical Properties

The mechanical properties of building materials are crucial in construction engineering, directly affecting the safety, durability, and service life of buildings. The strength, stiffness, and ductility of materials determine a building’s responsiveness to external loads (such as wind, earthquakes, and traffic), ensuring its stability and safety. Furthermore, excellent mechanical properties help materials maintain performance over the long term, reducing maintenance and replacement frequency and extending the building’s service life [76,77]. In terms of energy conservation and environmental protection, materials with excellent mechanical properties can reduce the number of materials required for construction, thereby reducing energy consumption and carbon emissions, and improving the economic efficiency and environmental friendliness of buildings. At the same time, mechanical properties affect the adaptability of materials across different environments, such as resistance to thermal expansion, corrosion, and seismic loading, and also influence construction efficiency and safety. Therefore, the mechanical properties of building materials not only relate to the structural safety of buildings but also determine their functionality, durability, and economic benefits in practical applications.

Zhong et al. [78] proposed and explored a novel PMMA/PVDF composite foam material that achieved an ultra-high expansion ratio of 120 times using CO_2_ foaming technology. The mechanical properties of this foam are particularly outstanding, with a negative Poisson’s ratio that gives it excellent compressive strength, elasticity, and flexibility. Even with a high expansion ratio, the foam material still has extremely high durability. In addition, the foam’s thermal conductivity is 26.69 mW/(m·K), which is significantly lower than that of traditional building materials, effectively reducing heat conduction in buildings and improving energy efficiency. The solar reflectance of the PMMA/PVDF composite foam is 96.37%, and its infrared emissivity is 97.34%, which can reduce the indoor temperature by 15 °C in high-temperature environments.

Wen et al. [79] proposed a silk fibroin-based thin film that significantly enhanced its mechanical properties and light transmittance through supramolecular crosslinking. The prepared Bolas-shaped polyethylene glycol peptide (BPP)/SF film had a tensile strength of 27.8 MPa, an elongation of up to 147%, and a toughness of 3.64 MJ/m^3^, demonstrating excellent mechanical strength and flexibility. The material also exhibited a mid-infrared (MIR) emissivity of 90.5%, demonstrating outstanding performance in passive radiative cooling.

Furthermore, to overcome the mechanical fragility of highly porous cellulosic materials, Liang et al. [80] developed a high-strength cellulose composite aerogel. Benefiting from its structurally reinforced design, the aerogel achieves an exceptional tensile strength of 6.9 MPa. In addition to its robust mechanical and flame-retardant properties, it exhibits a high solar reflectance of 93.4% and a mid-infrared emissivity of 97.4%. This synergistic combination enables approximately 7 °C of sub-ambient cooling under direct sunlight, providing a highly durable and efficient material platform for practical building applications.

## 4. Conclusions

In conclusion, radiative cooling materials represent a promising solution to the growing demand for energy-efficient building technologies. The integration of these materials into construction and building design can significantly reduce the reliance on energy-intensive air conditioning systems, mitigate the urban heat island effect, and contribute to sustainable urban development.

(1) Significant progress has been made in inorganic materials, resulting in a large number of daytime radiative cooling materials with promising applications. While these designs demonstrate high efficiency in radiation cooling, they are primarily manufactured on a small scale, requiring bulky casings and shielding. Furthermore, because most photonic materials are semi-transparent, they must be deposited on metallic mirrors to achieve high solar reflectivity. Therefore, the search for more direct methods and more scalable preparation of inorganic radiative cooling materials is essential.
(2) Petroleum-based polymer radiative cooling materials can be produced at a large scale when achieving good passive daytime cooling conditions. However, they suffer from lower mechanical properties, rapid aging, and shorter service life during application, while also causing environmental problems and further increasing primary energy consumption.
(3) The research demonstrates the application potential of cellulose and its derivatives as radiative cooling materials. While cellulose-based radiative cooling materials currently exhibit high sustainability, their cooling performance can still be improved through structural optimization. However, cellulose-based radiative cooling materials still face challenges, including balancing material strength and flexibility, and a lack of long-term stability in energy-efficient buildings.
(4) Radiative cooling materials require the mechanical properties, waterproofing, and long-term stability of traditional building materials. These properties can be improved through physical, chemical, biological, or radiation treatments. Furthermore, radiative cooling materials for buildings should have strong prospects for sustainable development, including minimizing their impact on the ecological environment and human development throughout raw material production, preparation, use, and subsequent treatment. Natural cellulose materials are renewable and biodegradable, making them ideal green building radiant cooling materials.
(5) Looking forward, the extensive rooftop applications of radiative cooling materials present immense opportunities for multi-functional integration. Beyond solely passive cooling, these materials can be strategically combined with active solar harvesting systems, such as photovoltaic (PV) panels and solar water heaters. Specifically, applying radiative cooling coatings to PV panels can dissipate excess heat and enhance photoelectric conversion efficiency. This synergistic integration of passive thermal management and active renewable energy technologies maximizes both space utilization and energy efficiency, paving the way for next-generation zero-energy buildings.

## Data Availability

No new data were created or analyzed in this study. Data sharing is not applicable to this article.
